# Review on the development of genotyping methods for assessing farm animal diversity

**DOI:** 10.1186/2049-1891-4-2

**Published:** 2013-01-23

**Authors:** Wanjie Yang, Xiaolong Kang, Qingfeng Yang, Yao Lin, Meiying Fang

**Affiliations:** 1 Department of Animal Genetics and Breeding, National Engineering Laboratory for Animal Breeding, MOA Laboratory of Animal Genetics and Breeding, College of Animal Science and Technology, China Agricultural University, Beijing, 100193, P. R. China; 2 College of Agriculture, Ningxia University, Yinchuan, 750021, P.R. China; 3 National Animal Husbandry and Veterinary Service, Beijing, 100094, P.R. China

**Keywords:** Farm animal genetic resources, Genetic evaluation, Genetic markers, Molecular markers

## Abstract

Advances in molecular biotechnology have introduced new generations of molecular markers for use in the genetic improvement of farm animals. Consequently, more accurate genetic information can be obtained to better understand existing animal genetic resources. This review gives a brief summary on the development of genetic markers including both the classical genetic markers and more advanced DNA-based molecular markers. This review will help us better understand the characteristics of different genetic markers and the genetic diversity of animal genetic resources.

## Introduction

The development of every species under its particular natural ecosystem, environmental, and socio-economic conditions has led to each having its own specific genetic characteristics. Collectively, these characteristics constitute the Earth’s species diversity. Mankind can learn and make use of these special genetic resources to develop animal production for human food needs. However, sufficient genetic markers for evaluating the population structure and other aspects of available animal genetic resources are necessary to assess genetic diversity.

In earlier studies, morphological markers and eco-geographical factors were used to represent diversity, and after that, chromosomal karyotyping was developed. With the rapid development of modern biotechnology, biochemical markers, such as proteins and isozymes, were utilized. By the 1980s, many different types of DNA molecular markers had been explored, e.g. Restriction Fragment Length Polymorphism (RFLP), Random Amplified Polymorphic DNA (RAPD), Amplified Fragment Length Polymorphism (AFLP), Single-Strand Conformation Polymorphism (SSCP) and Microsatellite DNA. All of these DNA-based markers contain specific advantages and have played significant roles in the evaluation of genetic diversity in farm animals. In addition, with biotechnological and computer innovations, novel strategies such as whole-genome SNP chips and DNA Barcoding have emerged. At present, DNA molecular marker techniques are widely applied in the fields of germplasm identification, phylogenetics, and genetic structural analysis. They overcome the limitations of morphological, cytological, and biochemical markers, namely the small numbers of such markers and the fact they can be environmentally influenced. The expansion in DNA information will facilitate study of genome-wide diversity; such information is much more precise for the assessment of genetic diversity than previous markers. The following is a brief summary on the principles and advancements of primary genetic markers involved in assessments of Animal Genetic Resources (AnGR).

### Conventional methods applied to AnGR assessments

#### Morphological markers

Morphological markers normally refer to external animal characteristics (i.e. coat color, body shape, skin structure, and anatomical characteristics) [1,2], which can be obtained by direct visual observation and measurement. They are used in the identification, classification, and characterization of genetic evolution of different species or populations. However, an animal’s phenotype is determined by its genetic background and the environment it experiences. The evaluation of farm animal genetic resources through morphological markers is based on subjective judgments and descriptions, and the conclusions reached are often not completely accurate. Furthermore, the measurement and identification of animal morphological traits usually takes a long time, and it is not easy to remove the effects of environmental factors. Consequently, the application of morphological markers is limited in the evaluation of quantitative traits. However, it is still an effective method for the assessment of qualitative traits, for which it is easy to characterize phenotypic differences between individuals through direct observation and measurement.

#### Cytological markers

Cytological markers have been used for the assessment of farm animal genetic resources [3,4] based on the numbers and morphology of animal chromosomes. Cytological markers include chromosome karyotypes, bandings, repeats, deletions, translocations, and inversions. Chromosomes are the carriers of genetic material and chromosome mutations are crucial sources of genetic variation [5], we can use these mutations as markers to determine the specific location of a gene on the chromosome, and its position relative to other genes. For instance, researchers can trace the origins and evolutionary history of livestock [6], and assess the genetic diversity of domesticated animals by comparing chromosome number and structure between domesticated animals and their wild ancestors [7].

#### Biochemical markers

Biochemical markers, e.g. blood type and isozymes, represent biochemical traits and can be analyzed by protein electrophoresis. In 1967, Buvanendran *et al*. investigated the genetic variation within species and phylogenetic relationships between species by differences in the amino acid composition of isozymes and soluble proteins [8]. Nevertheless, neither proteins nor isozymes are genetic material but the products of gene expression, and they are vulnerable to environmental impacts and individual growth discrepancies, limiting the breadth of their application [9]. Conversely, protein electrophoresis is a rapid, economic, and straightforward technique and provides a more detailed representation of polymorphisms than morphological or cytological markers; thus, it is still widely used in elucidating the origin and classification of species [10].

#### Molecular markers (DNA based markers)

With the development of molecular biotechnology, molecular markers have made rapid progress. A molecular marker is based on the nucleotide sequence mutations within the individual’s genome; they are the most reliable markers available. Molecular markers can be used for investigating genetic variations at the DNA level between different populations and individuals; its advantage is being able to find genetic variations rapidly and directly. Molecular markers have developed quickly, and they are becoming more and more informative. Up to now, various types of molecular markers have been utilized to evaluate DNA polymorphisms, e.g. RFLPs. Polymerase chain reaction (PCR) [11] can exponentially amplify a fragment of DNA *in vitro*, and since its invention a series of techniques have emerged in combination with PCR, e.g. PCR—RFLP, AFLP, simple sequences repeats (SSRs), and Single Nucleotide Polymorphisms (SNPs). In this review, we mainly focus on the introduction of several important DNA-based markers, and their various applications in characterizing animal genetic resources.

#### RFLP markers

RFLP is a method established by Grodzicker *et al*. in 1974, it is used to identify DNA polymorphisms among different individuals [12]. Its basic principle is as follows: first, genomic DNA from different individuals is digested into DNA fragments of varying size, using known restriction enzymes. Second, the digested fragments are separated via electrophoretic analysis. Finally, separated fragments are hybridized with radioactive or chemiluminescent homologous probes and exposed to an X-ray film; the different fragments are visible by autoradiography. The molecular basis of RFLP is that nucleotide base substitutions, insertions, deletions, duplications, and inversions within the whole genome can remove or create new restriction sites.

RFLP was the first DNA-based marker for constructing genetic linkage maps; it is also one of the most widely used markers in AnGR assessments and breeding program development. By combining this method with PCR (PCR-RFLP), Jiang and Gibson [13] detected four new genetic polymorphisms in the leptin gene of different pig breeds. The main advantages of RFLPs include: 1) high reliability, because it is generated from specific sites via known restriction enzymes and the results are constant over time and location. 2) Co-dominance, which means investigators are able to distinguish heterozygotes from homozygotes. 3) Selective neutrality refers to a situation in which different alleles of a certain gene confer equal fitness. The disadvantages of RFLPs are as follows: 1) labor-intensive and time-consuming. 2) RFLPs can only check out specific mutations at enzyme cut sites, which limits identification of whole genome variation in animals. 3) The polymorphism of RFLP markers is relatively low and must be detected by radioisotope, which limits its application.

#### RAPD markers

RAPD was developed by U.S. scientists in 1990 [14,15]. It amplifies the target genomic DNA with short, arbitrary primers (commonly 10 bp) in a PCR reaction, and can be used to produce relatively complicated DNA profiles for detecting amplified fragment length polymorphisms between organisms. Since the arbitrary primers complement different parts of the genomic DNA, PCR products will differ in number and size (polymorphism).

RAPD-PCR fingerprints have been successfully used in defining genetic diversity among different species. For example, the RAPD method was used to generate specific fingerprint patterns of ten different species: including wild boar, pig, horse, buffalo, beef, venison, dog, cat, rabbit, and kangaroo [16].

RAPD markers have several obvious features as summarized in the literature: 1) no prior sequence knowledge is necessary for designing the specific primers, which can then be used in different templates. 2) The amount of DNA required is very small because it will be amplified by PCR. 3) RAPDs are simple, quick, and cost effective compared to RFLP [17,18]. However, RAPDs also have some disadvantages, these include 1) the repeatability and reliability of RAPD polymorphic profiles are poor [19]. 2) Some non-specific and therefore non-reproducible binding of primers occurs. 3) RAPDs are dominant genetic markers which cannot be used to distinguish homozygote from heterozygote genotypes in F2 populations.

#### AFLP markers

AFLP was developed by Zabeau and Vos in 1993; it is a combination of the RFLP and PCR techniques [20]. The AFLP procedure is as follows: first, the genomic DNA is digested with a restriction enzyme, and then the digested fragments are ligated to synthetic adaptors and amplified with specified primers that are complementary to a selective sequence on the adaptors. Subsequent separation of the amplified fragments is obtained by selective primers and visualized using autoradiography [21]. AFLPs overcome the drawbacks of the labor-intensive, time-consuming RFLP method and solve the reliability problem caused by non-specific amplifications in RAPDs. Hoda *et al.* used AFLPs to assess genetic diversity and relationship among different breeds of sheep. They analyzed 93 unrelated individuals from three local Albanian sheep breeds markers. The results obtained indicated high diversity in Albania sheep breeds [22].

AFLPs are notable for their genetic stability, they provides an effective, rapid, and economical tool for detecting a large number of polymorphic genetic markers, that can be genotyped automatically [23,24]. However, AFLPs are dominant bi-allelic markers [23], and are unable to distinguish dominant homozygous from dominant heterozygous individuals [25]. The AFLP method is an ideal molecular approach for population genetics and genome typing, it is consequently widely applied to detect genetic polymorphisms, evaluate, and characterize animal genetic resources [26-29].

#### Microsatellite DNA markers

Microsatellite DNA, also known as simple sequences repeats (SSRs) or short tandem repeats (STRs), are common repeated sequences within eukaryotic genomes. Generally they consist of motifs which are made up of 1–6 base pairs (bp) tandemly repeated several times (e.g. CACACACACACACACA) [30,31]. The flanking regions of repeated sequences at microsatellite loci are mostly conservative and the repetition motifs are highly variable between different species and even different individuals of the same species. So we can design specific primers based on the conserved sequences and amplify the core repeat sequences by way of PCR, genetic polymorphisms can then be detected via electrophoresis [31].

SSRs have the same advantages as RFLPs, and avoid the utilization of radioisotopes essential for RFLPs; it has higher repeatability and stability than RAPDs; compared to AFLP markers, SSRs are co-dominant markers and able to distinguish homozygotes from heterozygotes. Until recently, microsatellites were the markers most widely used for genetic diversity, mapping quantitative trait loci for production, and functional traits in farm animals [32-34]; they have also been used for marker assisted selection practices [35].

The advantages and disadvantages of SSR markers have been reported by many authors [36-40]. Its advantages are as follows: low quantities of template DNA required (10–100 ng), high polymorphism, co-dominant markers, high accuracy, high reproducibility, different microsatellites can be multiplexed in PCR, and they are amenable to automation. Its disadvantages include: time-consuming and expensive to develop, heterozygotes may be misclassified as homozygotes when null-alleles occur because of mutations in the primer annealing sites, stutter bands may complicate accurate scoring of polymorphisms, underlying mutation model largely unknown, and microsatellite markers do help to identify neutral biodiversity but do not provide information on functional trait biodiversity. Despite these disadvantages, microsatellite markers are still popular nuclear DNA markers for the investigation of genetic variation among and within species.

### New approaches for AnGR assessments

In addition to the classical markers discussed above, with the development of modern molecular techniques and the completion of the Human Genome Project (HGP), some new markers have emerged and are being used in the evaluation of farm animal genetic resources; these include high-density SNP arrays, whole-genome sequencing, and DNA barcoding.

#### SNP markers and whole-genome sequencing

SNP, a novel molecular marker technology, was first proposed by Lander in 1996, it refers to a sequence polymorphism caused by a single nucleotide mutation at a specific locus in the DNA sequence. This sort of polymorphism includes single base transitions, transversions, insertions and deletions [41], and the minor allele frequency should be 1% or greater [42]. Of all the SNP mutation types, transitions are the most common (approx. 2/3) [43]. Currently, SNP markers are one of the preferred genotyping approaches, because they are abundant in the genome, genetically stable, and amenable to high-throughput automated analysis [42].

The fundamental principle of SNPs is to hybridize detected DNA fragments with high-density DNA probe arrays (also called SNP chips); the SNP allele is then named according to the hybridization results. SNPs are bi-allelic markers, indicating a specific polymorphism in only two alleles of a population [44]. SNPs distribute in both coding and non-coding regions of genomes, they are vital players in the process of population genetic variations and species evolution [45].

Currently, DNA chip technology is usually carried out during SNP investigations. A group of associated SNP loci located on a certain region of the chromosome can form one SNP haplotype. SNPs are third generation molecular marker technology coming after RFLPs and SSRs [46]; it has been successfully used to investigate genetic variation among different species and breeds [47-49].

Compared with previous markers, SNPs have the following advantages: 1) they are numerous and widely distributed throughout the entire genome [50]. 2) High genetic stability, excellent repeatability, and high accuracy. 3) Allow for fast, high-throughput genotyping [51]. 4) Convenient for effectively distinguishing heterozygote from homozygote alleles because of its co-dominances.

Because of their extensive distribution and abundant variations, SNPs play an important role in farm animal population structure, genetic differentiation, origin, and evolution research. For example, linkage disequilibrium (LD) among different SNPs can be utilized for association analysis. Furthermore, we can gain information concerning animal population diversity and population evolution (origins, differentiation, and migrations) via SNP haplotypes among different populations.

One disadvantage of SNP markers is the low level information obtained compared with that of a highly polymorphic microsatellite, but this can be compensated for by employing a higher numbers of markers (SNP chips) and whole-genome sequencing [52,53].

With the improvement of sequencing technology, whole-genome/gene sequencing has become available for characterizing genetic diversity among farm animals. It is the most straight-forward method and provides more complete information on the genetic variation among different populations because it can detect all the variations within the genome. Currently, the problem with whole-genome sequencing is setting up a high-through data analysis platform to explore useful information for the conservation and utilization of farm animals.

#### DNA barcoding markers

Barcoding is an automatic scanning and identification technology, which has emerged from practical computer technologies. Biological taxonomists apply this principle to species classification, referring to a DNA barcode. A DNA barcode is a Short DNA sequence from a standardized region of the genome used for identifying species. The intent of DNA barcoding is to use large-scale screening of one or more reference genes in order to (i) assign unknown individuals to species, and (ii) enhance discovery of new species [54,55].

Tautz *et al*. [56] were the first researchers to use the DNA sequences in systematical biological taxonomy (also called DNA taxonomy). Subsequently, Hebert *et al*. [54] proposed the concept of DNA Barcoding and suggested its use for a single mtDNA gene, mitochondrial cytochrome *c* oxidase I (COI), as a common sequence in animal DNA barcoding studies. Researchers can compile a public library of DNA barcodes linked to named specimens, which can provide a new master key for identifying species diversity [57].

Compared with time-consuming and inefficient traditional morphological classification [58], DNA Barcoding has a high accuracy of 97.9% [59], and provides us a new, quick, and convenient identification strategy for animal genetic diversity [54]. However, as with the other markers mentioned the DNA barcoding technique also has some disadvantages: 1) the genome fragments are very difficult to obtain and are relatively conservative and have no enough variations. Some organisms cannot be identified with COI because of the low evolution rates of COI sequences in some species. 2) COI is an mtDNA sequence of maternal origin, which could bias species diversity [60,61]. The above disadvantages can be compensated for by using one or more nuclear gene barcodes together to make a standardized analysis of AnGR.

## Summary

Farm animals are extremely important to humans, supplying some 30% of our total food requirements [62]. The accurate evaluation of animal genetic resources is the basis for their conservation and utilization. From the first demonstration of RFLPs to the current whole-genome sequencing, many methods have been developed and tested at the DNA sequence level, providing a large number of markers and opening up new opportunities for evaluating diversity in farm animal genetic resources. Currently, SSR and SNP markers are valuable tools for evaluating germplasm diversity because of their high EMI (effective marker index) and high QND (qualitative nature of data), respectively [63]. With the development of new markers, more accurate genetic evaluation is possible. The development of molecular markers will continue in the near future and provide better understanding of animal genetic resources.

## Competing interests

The authors declare that they have no competing interests.

## Authors’ contributions

WY, XK, QY and MF contributed to the writing of this review paper. All authors read and approved the final manuscript.

## References

[B1] Van Wezel IL Rodgers RJ Morphological characterization of bovine primordial follicles and their environment in vivo Biol Reprod 1996 55 1003 1011 10.1095/biolreprod55.5.1003 8902210

[B2] Gizaw S Van Arendonk JAM Komen H Windig JJ Hanotte O Population structure, genetic variation and morphological diversity in indigenous sheep of Ethiopia Anim Genet 2007 38 621 628 10.1111/j.1365-2052.2007.01659.x 18028516

[B3] Nadler CF Hoffmann RS Woolf A G-band patterns as chromosomal markers, and the interpretation of chromosomal evolution in wild sheep (Ovis) Cell Mol Life Sci 1973 29 117 119 10.1007/BF01913288 4125751

[B4] Popescu NC Evans CH DiPaolo JA Chromosome patterns (G and C Bands) of in vitro chemical carcinogen-transformed guinea pig cells Cancer Res 1976 36 1404 1260765

[B5] Bitgood JJ Shoffner RN Cytology and cytogenetics Poultry breeding Genet 1990 22 401 427

[B6] Silversides FG Crawford RD Wang HC The cytogenetics of domestic geese Heredity 1988 79 6 8 10.1093/oxfordjournals.jhered.a110449 3367038

[B7] Becak ML Becak W Roberts FL Fish, amphibians, reptiles and birds 1973 Springer-Verlag, Berlin, Heidelberg, New York

[B8] Buvanendran V Finney DJ Linkage relationships of egg albumen loci in the domestic fowl Br Poult Sci 1967 8 9 13 10.1080/00071666708415644

[B9] Drinkwater RD Hetzel DJS Application of molecular biology to understanding genotype-environment interactions in livestock production Proc. of an International Symposium on Nuclear Techniques in Animal Production and Health 1991 IAEA, FAO, Vienna 437 452 15–19 April

[B10] Jonker J Meurs G Balner H Typing for RhLA-D in rhesus monkeys: II. genetics of the D antigens and their association with DR antigens in a population of unrelated animals Tissue Antigens 1982 19 69 78 10.1111/j.1399-0039.1982.tb01417.x 7071818

[B11] Mullis K Faloona F Scharf S Saiki R Horn G Erlich H Specific enzymatic amplification of DNA In vitro: the polymerase chain reaction Cold Spring Harb Symp Quant Biol 1986 51 263 273 10.1101/SQB.1986.051.01.032 3472723

[B12] Grodzicker T Williams J Sharp P Physical mapping of temperature-sensitive mutations of adenovirus Cold Spring Harbor Symp Quant Biol 1974 34 439 446 10.1101/sqb.1974.039.01.056 169087

[B13] Jiang ZH Gibson JP Genetic polymorphisms in the leptin gene and their association with fatness in four pig breeds Mammal Genome 1999 10 191 193 10.1007/s003359900968 9922403

[B14] Williams JGK Kubeilik AR Livak KJ Rafalski JA Tingey SV DNA polymorphisms amplified by arbitrary primers are useful as genetic markers Nucleic Acids Res 1990 18 6531 6535 10.1093/nar/18.22.6531 1979162 PMC332606

[B15] Welsh J McClelland M Fingerprinting genomes Using PCR with arbitrary primers Nucleic Acids Res 1990 18 7213 7218 10.1093/nar/18.24.7213 2259619 PMC332855

[B16] Koh MC Lim CH Chua SB Chew ST Phang STW Random amplified polymorphic DNA (RAPD) fingerprints for identification of red meat animal species Meat Sci 1998 48 275 285 10.1016/S0309-1740(97)00104-6 22063076

[B17] Demeke T Adams RP Chibbar R Potential taxonomic use of random amplified polymorphic DNA (RAPD): a case study in Brassica Theor Appl Genet 1992 84 990 994 10.1007/BF00227415 24201505

[B18] Koller B Lehmann A McDermott JM Identification of apple cultivars using RAPD markers Theor Appl Genet 1993 85 901 904 10.1007/BF00225036 24196067

[B19] Meunier JR Grimont PAD Factors affecting reproducibility of random amplified polymorphic DNA fingerprinting Res Microbiol 1993 144 373 379 10.1016/0923-2508(93)90194-7 8248630

[B20] Zabeau M Vos P Selective restriction fragment amplification: a general method for DNA fingerprinting EP Patent 1993 0534858 B2

[B21] Blears MJ De Grandis SA Lee H Trevors JT Amplified fragment length polymorphism (AFLP): a review of the procedure and its applications J Indus Micro Biotech 1998 21 99 114 10.1038/sj.jim.2900537

[B22] Hoda A Ajmone-Marsan P Dobi P Bozgo V Consortium E Genetic diversity in Albanian sheep breeds estimated by AFLP markers Alban J Agricul Sci 2010 9 23 29

[B23] Vos P Hogers R Bleeker M Reijans M Lee TVD Hornes M Friters A Pot J Paleman J Kuiper M Zabeau M AFLP: a new technique for DNA fingerprinting Nucleic Acids Res 1995 23 4407 4414 10.1093/nar/23.21.4407 7501463 PMC307397

[B24] Vos P Kuiper M Caetano-Anollés G, Gresshoff PM AFLP analysis, in DNA markers: protocols, applications, and overviews 1997 115 131

[B25] Paglia G Morgante M PCR-based multiplex DNA fingerprinting technique for the analysis of conifer genome Mol Breed 1998 4 173 177 10.1023/A:1009637608702

[B26] Ajmone-Marsan P Negrini R Milanesi E Bozzi R Nijman IJ Buntjer JB Valentini A Lenstra JA Genetic distances within and across cattle breeds as indicated by biallelic AFLP markers Anim Genetics 2002 33 280 286 10.1046/j.1365-2052.2002.00865.x 12139507

[B27] Ajmone-Marsan P Negrini R Milanesi E Colli L Pellecchia M Nicoloso L Crepaldi P Lenstra JA Breed assignment of Italian cattle using biallelic AFLP-markers Anim Genet 2007 38 147 153 10.1111/j.1365-2052.2007.01573.x 17326802

[B28] Negrini R Milanesi E Bozzi R Pellecchia M Ajmone-Marsan P Tuscany autochthonous cattle breeds: an original genetic resource investigated by AFLP markers J Anim Breed Genet 2006 123 10 16 10.1111/j.1439-0388.2006.00554.x 16420260

[B29] Negrini R Nijmanl IJ Milanesi E Moazami-Goudarzi K Williams JL Erhardt G Dunner S Rodellar C Valentini A Bradley DG Olsaker I Kantanen J Ajmone-Marsan P Lenstra JA the European Cattle Genetic Diversity Consortium Differentiation of European cattle by AFLP fingerprinting Anim Genet 2007 38 60 66 10.1111/j.1365-2052.2007.01554.x 17257190

[B30] Litt M Luty JA A hypervariable microsatellite revealed by in vitro amplification of a dinucleotide repeat within the cardiac muscle actin gene Am J Hum Genet 1989 44 397 401 2563634 PMC1715430

[B31] Tautz D Hypervariability of simple sequences as a generalsource for polymorphic DNA markers Nucleic Acids Res 1989 17 6463 6471 10.1093/nar/17.16.6463 2780284 PMC318341

[B32] Fang M Braunschweig M Hu X Hu L Feng J Li N Wu C Genetic variation of exon 2 of SLA-DQBgene in Chinese pigs Biochem Genet 2005 43 119 125 10.1007/s10528-005-1504-3 15932061

[B33] Fang M Larson G Soares Ribeiro H Li N Andersson L Contrasting mode of evolution at a coat color locus in wild and domestic pigs PLoS Genet 2009 5 e1000341 10.1371/journal.pgen.1000341 19148282 PMC2613536

[B34] Hiendleder S Hiendleder S Thomsen H Reinsch N Bennewitz J Leyhe-Horn B Looft C Xu N Medjugorac I Russ I Kühn C Brockmann GA Blümel J Brenig B Reinhardt F Reents R Averdunk G Schwerin M Förster M Kalm E Erhardt G Mapping of QTL for body conformation and behavior in cattle J Heredity 2003 94 496 506 10.1093/jhered/esg090 14691316

[B35] Montaldo HH Meza-Herrera CA Use of molecular markers and major genes in the genetic improvement of livestock Elec J Biotech 1998 1 1 7

[B36] Bishop MD Kappes SM Keele JW Stone RT Sunden SL Hawkins GA Toldo SS Fries R Grosz MD Yoo J A genetic linkage map for cattle Genetics 1994 136 619 639 7908653 10.1093/genetics/136.2.619 PMC1205813

[B37] Bishop MD Hawkins GA Keeler CL Use of DNA markers in animal selection Ther 1995 43 61 70 10.1016/0093-691X(94)00018-P

[B38] Baron EE Mário LM Verneque RS Coutinho LL Parentage testing and effect of misidentification on the estimation of breeding value in Gir cattle Genetics and Mol Biol 2002 25 389 394

[B39] Mburu D Hanotte O A practical approach to microsatellite genotyping with special reference to livestock population genetics ILRI Biodiversity project: A manual prepared for the IAEA/ILRI training course on molecular characterisation of small ruminant genetic resources of Asia, October- December 2005 ILRI, Nairobi, Kenya

[B40] Erhardt E Weimann C Use of molecular markers for evaluation of genetic diversity and in animal production Arch Latinoam Prod Anim 2007 15 63 66

[B41] Lander ES The new genomics: global views of biology Science 1996 274 536 539 10.1126/science.274.5287.536 8928008

[B42] Vignal A Milan D SanCristobal M A review on SNP and other types of molecular markers and their use in animalgenetics Genet Selec Evol 2002 34 275 305 10.1186/1297-9686-34-3-275 PMC2705447 12081799

[B43] Zhao ZM Boerwinkle E Neighboring-nucleotide effects on single nucleotide polymorphisms: a study of 2.6 million polymorphisms across the human genome Genome Res 2002 12 1679 1686 10.1101/gr.287302 12421754 PMC187558

[B44] Kruglyak L The use of a genetic map of biallelic markers in linkage studies Nature Genet 1997 17 21 24 10.1038/ng0997-21 9288093

[B45] Syvänen AC Accessing geneic variation: genotyping single nucleotide polymorphisms Nature Rev Genet 2001 2 930 942 10.1038/35103535 11733746

[B46] Peter G An assessment of the utility of single nucleotide polymorphisms (SNPs) for forensic purposes Int J Legal Med 2001 114 204 210 10.1007/s004149900117 11355396

[B47] The Bovine HapMap Consortium Genome-wide survey of SNP variation uncovers the genetic structure of cattle breeds Science 2009 324 528 532 19390050 10.1126/science.1167936 PMC2735092

[B48] Brooks SA Gabreski N Miller D Brisbin A Brown HE Streeter C Mezey J Cook D Antczak DF Whole-genome SNP association in the horse: identification of a deletion in myosin Va responsible for lavender foal syndrome PLoS Genet 2010 6 e1000909 10.1371/journal.pgen.1000909 20419149 PMC2855325

[B49] Amaral AJ Megens HJ Crooijmans RPMA Heuven HCM Groenen MAM Linkage disequilibrium decay and haplotype block structure in the pig Genetics 2008 179 569 579 10.1534/genetics.107.084277 18493072 PMC2390633

[B50] Primmer CR Borge T Lindell J Single-nucleotidepolymorphism characterization in species withlimited available sequence information: high nucleotidediversity revealed in the avian genome Mol Ecol 2002 11 603 612 10.1046/j.0962-1083.2001.01452.x 11918793

[B51] Tsuchihashi Z Dracopoli NC Progress in high-throughput SNP genotyping methods J Pharmacogenomics 2002 2 103 110 10.1038/sj.tpj.6500094 12049172

[B52] Werner M Sych M Herbon N Illig T Konig IR Wjst M Large-scale determination of SNP allele frequencies in DNA pools using MALDI-TOF mass spectrometry Hum Mutat 2002 20 57 64 10.1002/humu.10094 12112658

[B53] Werner M Herbon N Gohlke H Altmuller J Knappw M Heinrich J Wjst M Asthma is associated with single-nucleotide polymorphisms in ADAM33 Clin Exp Allergy 2004 34 26 31 10.1111/j.1365-2222.2004.01846.x 14720258

[B54] Hebert PDN Cywinska A Ball SL de Waard JR Biological identifications through DNA barcodes Proc R Soc Biol Sci 2003 270 313 321 10.1098/rspb.2002.2218 PMC1691236 12614582

[B55] Stoecklem Taxonomy, DNA, and the Bar Code of Life BioScience 2003 53 796 797 10.1641/0006-3568(2003)053[0796:TDATBC]2.0.CO;2

[B56] Tautz D Arctander P Minelli A Thomas RH DNA points the way ahead in taxonomy Nature 2002 418 479 12152050 10.1038/418479a

[B57] Hebert PDN Penton EH Burns JM Ten species in one: DNA barcoding reveals cryptic species in theneotropical skipper butterfly Astraptesfulgerator Proc Nat Acad Sci USA 2004 101 14812 14817 10.1073/pnas.0406166101 15465915 PMC522015

[B58] Huang J Xu Q Sun ZJ Identifying earthworms through DNA barcodes Pedobiologia 2007 51 301 309 10.1016/j.pedobi.2007.05.003

[B59] Hajibabaei M Janzen DH Burns JM DNA barcodes distinguish species of tropical Lepidoptera Proc Nat Acad Sci USA 2006 103 968 971 10.1073/pnas.0510466103 16418261 PMC1327734

[B60] Meyer CP Paulay G DNA barcoding: error rates based on comprehensive sampling PLoS Biol 2005 3 2229 2238 10.1371/journal.pbio.0030422 PMC1287506 16336051

[B61] Meier R Shiyang K Vaidya G DNA barcoding andtaxonomy in Diptera: a tale of high intraspecific variability and lowidentification sucess Syst Biol 2006 55 715 728 10.1080/10635150600969864 17060194

[B62] LID (Livestock in Development) Livestock in poverty-focused development 1999 Livestock in Development, Crewkerne, UK

[B63] Varshney RK Chabane K Hendre PS Aggarwal RK Graner A Comparative assessment of EST-SSR, EST-SNP and AFLP markers for evaluation of genetic diversity and conservation of genetic resources using wild, cultivated and elite barleys Plant Sci 2007 173 638 649 10.1016/j.plantsci.2007.08.010

